# Cell Wall Epitopes and Endoploidy as Reporters of Embryogenic Potential in *Brachypodium Distachyon* Callus Culture

**DOI:** 10.3390/ijms19123811

**Published:** 2018-11-29

**Authors:** Alexander Betekhtin, Magdalena Rojek, Katarzyna Nowak, Artur Pinski, Anna Milewska-Hendel, Ewa Kurczynska, John H. Doonan, Robert Hasterok

**Affiliations:** 1 Department of Plant Anatomy and Cytology, Faculty of Biology and Environmental Protection, University of Silesia in Katowice, Katowice 40-007, Poland; magdalena.rojek@us.edu.pl (M.R.); apinski@us.edu.pl (A.P.); robert.hasterok@us.edu.pl (R.H.); 2 Department of Genetics, Faculty of Biology and Environmental Protection, University of Silesia in Katowice, Katowice 40-007, Poland; katarzyna.nowak@us.edu.pl; 3 Department of Cell Biology, Faculty of Biology and Environmental Protection, University of Silesia in Katowice, Katowice 40-007, Poland; anna.milewska@us.edu.pl (A.M.-H.); ewa.kurczynska@us.edu.pl (E.K.); 4 National Plant Phenomics Centre, IBERS, Aberystwyth University, Aberystwyth SY23 3EE, UK; john.doonan@aber.ac.uk

**Keywords:** arabinogalactan proteins, *Brachypodium distachyon*, cell wall, cyclins, extensins, pectins, ploidy instability, somatic embryogenesis, somaclonal variation, transcript accumulation

## Abstract

Effective regeneration of callus tissue into embryos and then into whole plants is essential for plant biotechnology. The embryonic potential is often low and can further decrease with time in culture, which limits the utilisation of calli for transformation procedures and in vitro propagation. In this study, we show that the loss of embryogenic potential in callus cultures of *Brachypodium distachyon* is progressive over time. Flow cytometry analyses indicated endoploidy levels increased in 60- and 90-day-old calli with effective loss of the 2C DNA content peak in the latter. Analysis of indolic compounds content revealed a decrease in 60- and 90-day-old calli compared to either freshly isolated explants or 30-day-old calli. Immunohistochemical analysis revealed a decrease in arabinogalactan proteins (AGP) signal with the time of culture, but extensin (EXT) epitopes either increased (JIM12 epitopes) or decreased (JIM11 epitopes). The transcript accumulation levels of *AGPs* and *EXTs* confirmed these results, with most of *AGP* and *EXT* transcripts gradually decreasing. Some chimeric *EXT* transcripts significantly increased on the 30th day of culture, perhaps because of an increased embryogenic potential. Selected somatic embryogenesis-related genes and cyclins demonstrated a gradual decrease of transcript accumulation for *YUCCA (YUC*), *AINTEGUMENTA-LIKE* (*AIL*), *BABY BOOM* (*BBM*), and *CLAVATA* (*CLV3*) genes, as well as for most of the cyclins, starting from the 30th day of culture. Notably, *WUSCHEL* (*WUS*) transcript was detectable only on the 30th and 60th day and was not detectable in the zygotic embryos and in 90-day-old calli.

## 1. Introduction

Somatic embryogenesis (SE) occurs when a single somatic cell or group of somatic cells, usually induced by external conditions of in vitro culture, changes its developmental programme and starts forming totipotent embryogenic cells capable of becoming complete plants [1]. Each stage of the process, from induction to seedling formation, is influenced by many different genetic, epigenetic, biochemical, and physiological factors. The correct progression of SE is controlled by a large group of transcription factors, e.g., *BABY BOOM* (*BBM*), *WUSHEL* (*WUS*), *LEAFY COTYLEDON1* (*LEC1*) and *LEAFY COTYLEDON2* (*LEC2*), *FUSCA3* (*FUS3*), proteins associated with signal transduction pathways, among which major groups are SOMATIC EMBRYOGENESIS RECEPTOR KINASE (SERK proteins) and CLAVATA proteins (CLV1, 2 and 3), as well as proteins involved in the regulation of hormone biosynthesis and response, for example, YUCCA1 (YUC1). These proteins are involved, variously, in dedifferentiation, promotion, and maintenance of cell totipotency and embryogenic cell formation [1,2,3,4,5]. Somatic embryo development may also require spatio-temporal control of cell division and elongation [6]. The eukaryotic cell cycle is controlled by the large family of *CYCLIN*-*DEPENDENT KINASES* (*CDKs*). The activity of these kinases is positively regulated by binding to cyclins, some of which appear and disappear periodically during the cell cycle, and their gene expression is often modulated during the progression of SE [7,8].

The switch from a somatic to an embryogenic state also involves changes in the cell wall composition and architecture [9,10,11]. Cell wall components, such as hemicelluloses, pectins, and arabinogalactan proteins (AGPs) undergo significant changes during initiation and progression of SE [11,12,13], and some provide diagnostic markers for SE [10].

A high regeneration ability (or morphogenetic potential) of callus tissue facilitates many biotechnological applications. However, the loss of morphogenetic potential is a common phenomenon that can occur soon after callus initiation or even as late as after many years of culture [14,15,16,17]. In any case, poor regeneration is a common problem that restricts the application of biotechnological manipulations in many species. The mechanisms by which a tissue loses its regeneration ability is therefore of general interest.

Sequential subculture and prolonged culture may induce various deleterious changes in genome and epigenome organisation, cell structure, transcription and physiology. Changes at the genomic level can lead to the regeneration of phenotypically variable or even abnormal plants. This phenomenon, known as somaclonal variation (SV), is common in long-term in vitro culture. Several factors, such as, in particular, the source of the initial explant, media composition, exogenous hormones, and the culture conditions can all affect SV. A frequently observed cytological change associated with SV is chromosome aberration, which can involve changes in ploidy and structural chromosome rearrangements [18]. In addition to genetic changes in regenerated plants, prolonged culture can cause the accumulation of point mutations leading to the reduction of the morphogenic potential of the callus tissue [19,20,21]. For example, tissue culture of *Quercus rubra* and *Catharantus roseus* are characterised by the rapid loss of morphogenic potential [2,22,23]. The embryogenic calli of *Fagopyrum tataricum* can maintain a high level of genome stability up to 10 years [14]. The differences between morphogenic and non-morphogenic calli of *F. tataricum* may be related to greater genomic stability of the morphogenic ones. A long-term callus line of *Lotus corniculatus* maintained by regular subculture for 1.5–2.5 years had a relatively high regenerative capacity, but the production of shoots decreased as the callus line grew older [24].

In this work, we evaluated a number of processes that could be related to the gradual loss of embryogenic potential in the callus culture of *Brachypodium distachyon*. We used histological and immunolocalisation techniques to analyse the distribution of selected pectins, AGPs, extensins (EXTs), and hemicelluloses in the cell walls and internal cell compartments and on the embryogenic callus surface. Flow cytometry was used to follow how the nuclear DNA content changed with the time of culture. The transcript accumulation level of selected genes during the time course was also determined. It may be possible to develop one or more of these markers as a diagnostic tool to identify embryogenic calli or assess calli’s overall regenerative potential.

## 2. Results

### 2.1. Changes in Morphological, Histological, and Biochemical Features of B. Distachyon Calli during their Gradual Loss of Embryogenic Potential

**Figure 1:**
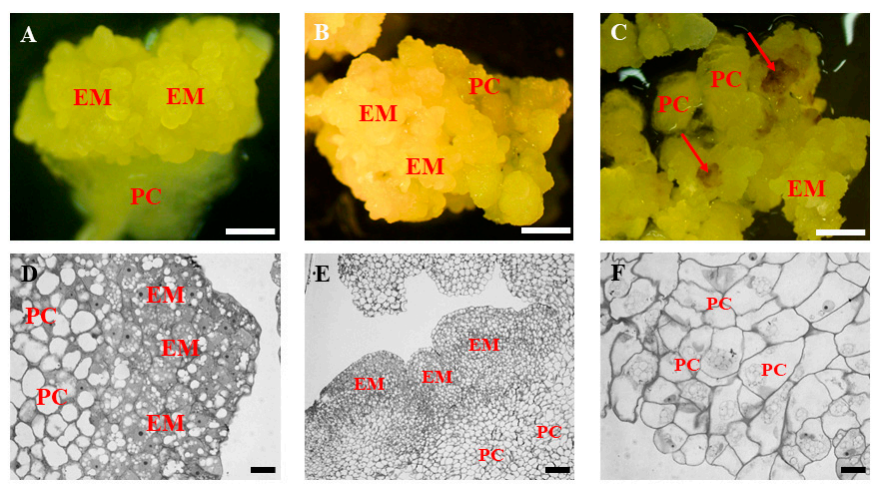
Morphology and histology of representative structures in the in vitro culture of *Brachypodium distachyon* callus. (**A**–**C**) General morphology and (**D**–**E**) histological section of the callus on the 30th (**A**,**D**), 60th (**B**,**E**), and 90th (**C**,**F**) day of cultivation. The red arrows point at brownish parts of the callus. EM: embryogenic masses, PC: parenchymatous cells. Scale bars, (**A**–**C**)—1 mm, (**D**)—10 μm, (**E**)—50 μm, (**F**)—10 μm.

In general, embryogenic calli on the 30th and 60th day of culture were either vitreous and friable or compact and composed of embryogenic masses that were yellowish in colour (Figure 1A,B). On the 90th day of culture, the callus was mostly vitreous and friable with some brownish parts (Figure 1C, red arrows). Histological analyses of 30- and 60-day-old calli revealed the presence of two cell types. i.e., embryogenic cells that were located at the callus surface and parenchymatous cells that were located inside the callus (Figure 1D,E). The embryogenic cells were characterised by the presence of several small vacuoles, dense cytoplasm, and a well-defined nucleus containing a distinct nucleolus. In contrast, the parenchymatous cells were larger and highly vacuolated, and their nuclei were located in the periphery next to the cell wall. On the 90th day of culture, the calli were characterised by the presence of mostly parenchymatous cells (Figure 1F) with few embryogenic-type cells.

**Figure 2:**
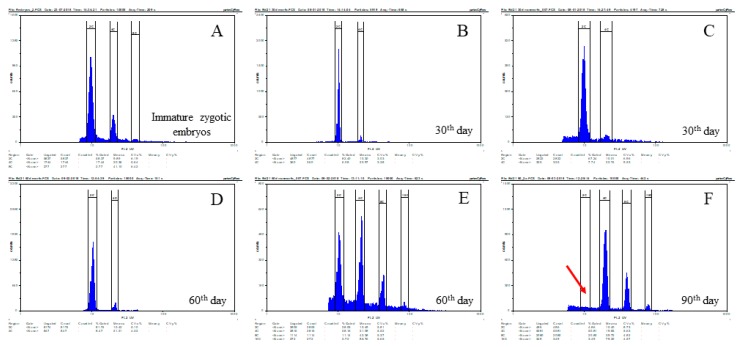
Relative DNA content determined by flow cytometry in zygotic embryos (**A**) and in *B. distachyon* callus on the 30th (**B**–**C**, embryogenic and non-embryogenic parts, respectively), 60th (**D**–**E**, embryogenic and non-embryogenic parts, respectively), and 90th (**F**) day of cultivation. The red arrow demonstrates the absence of 2C DNA on the 90th day of cultivation.

Flow cytometry analyses revealed significant differences in the nuclear DNA content between calli at different times of culture (Figure 2). The zygotic embryo was usually characterised by the presence of nuclei with 2C and 4C DNA content, but a small peak representing nuclei with 8C DNA content was also present (Figure 2A). Separate analyses of embryogenic masses and non-embryogenic parts of 30-day-old calli demonstrated similar patterns of relative DNA contents in both parts, with 2C and 4C nuclei predominating (Figure 2B–D). However, parenchymatous cells from 60-day-old calli were significantly different, with clear peaks of DNA content ranging from 2C to 16C clearly observed (Figure 2E). In 90-day-old calli, in which it was difficult or impossible to distinguish different cell types, flow cytometry analysis revealed the presence of peaks representing 4C, 8C, and 16C DNA content (Figure 2E). The absence of a detectable 2C DNA content peak in 90-day-old calli was noted (Figure 2F, red arrow).

**Figure 3:**
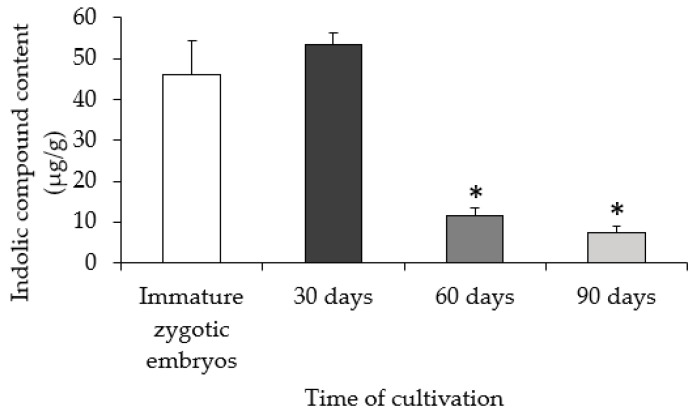
Indolic compounds content on the 30th, 60th, and 90th day of cultivation. The asterisks * indicate values that are significantly different from the immature zygotic embryo control (Student’s *t*-test, *p* < 0.05; *n* = 3 ± SD).

Analysis of indolic compounds in the explant and embryogenic calli revealed a gradual decrease of these molecules during culture (Figure 3). The highest contents (46.3 and 53.2 µg/g) were found in freshly isolated explants and in 30-day-old calli, respectively. On the 60th and 90th day of culture, the calli had the lowest content of indolic compounds (11.6 and 8.8 µg/g, respectively).

### 2.2. Localisation and Transcript Accumulation Level Analyses of Hydroxyproline-Rich Proteins (HRGPs), Pectins, and Hemicelluloses

To test whether selected hydroxyproline-rich protein (HRGPs), pectin, and hemicellulose epitopes were differently localised in callus cells on days 30 and 90, (highest and lowest embryogenic potential, respectively), immunocytochemical analyses were performed using the following monoclonal antibodies: JIM13, JIM16, and LM2 against specific cell wall epitopes such as AGPs and EXTs (JIM11 and JIM12), LM6, LM16, LM19, LM20 against pectins, and LM25 against hemicelluloses. The epitopes recognised by the monoclonal antibodies and relevant references are provided in Table 1.

**Figure A1:**
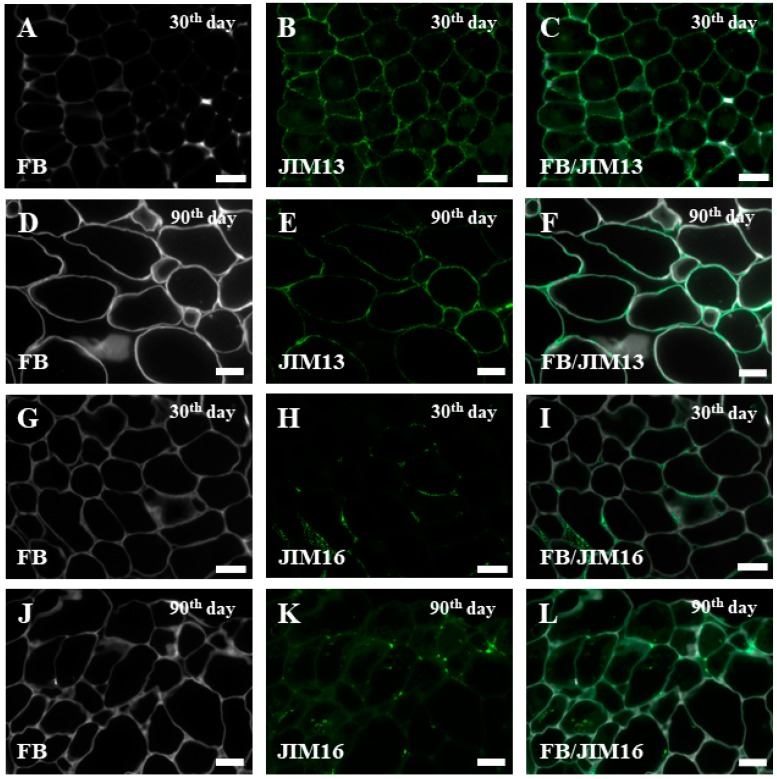
Immunolocalisation of JIM13 (**A**–**F**) and JIM16 (**G**–**L**) in *B. distachyon* callus. (**A**–**C**, **G**–**I**) 30th day of cultivation; (**D**–**F**, **J**–**L**) 90th day of cultivation. **FB:** fluorescent brightener. Scale bars, 10 μm.

**Figure A2:**
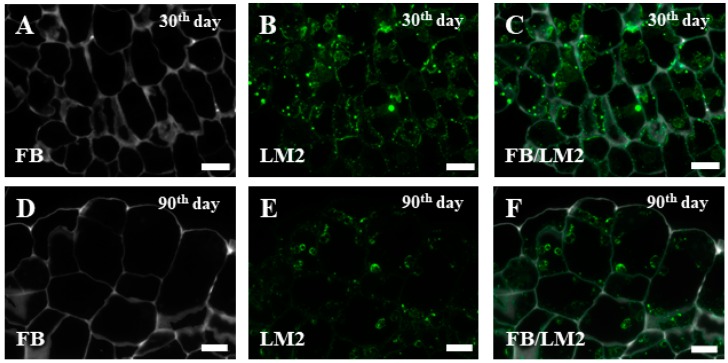
Immunolocalisation of LM2 (**A**–**F**) in *B. distachyon* callus. (**A**–**C**) 30th day of cultivation; (**D**–**F**) 90th day of cultivation. Scale bars, 10 μm.

**Figure A3:**
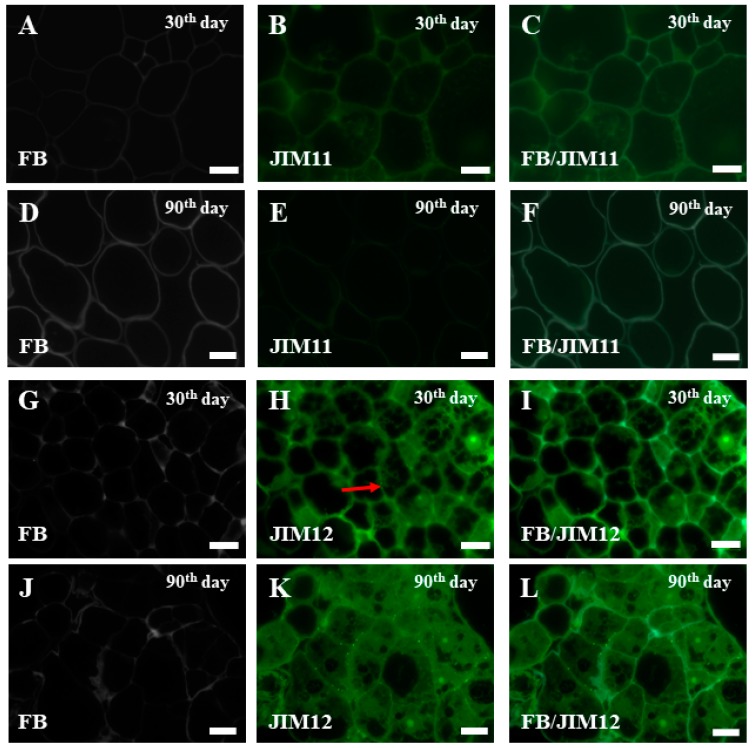
Immunolocalisation of JIM11 (**A**–**F**) and JIM12 (**G**–**L**) in *B. distachyon* callus. (**A**–**C**, **G**–**I**) 30th day of cultivation; (**D**–**F**, **J**–**L**) 90th day of cultivation. The red arrow indicates a very weak signal for JIM12. The greenish background on the photomicrographs is due to autofluorescence. Scale bars, 10 μm.

**Figure A4:**
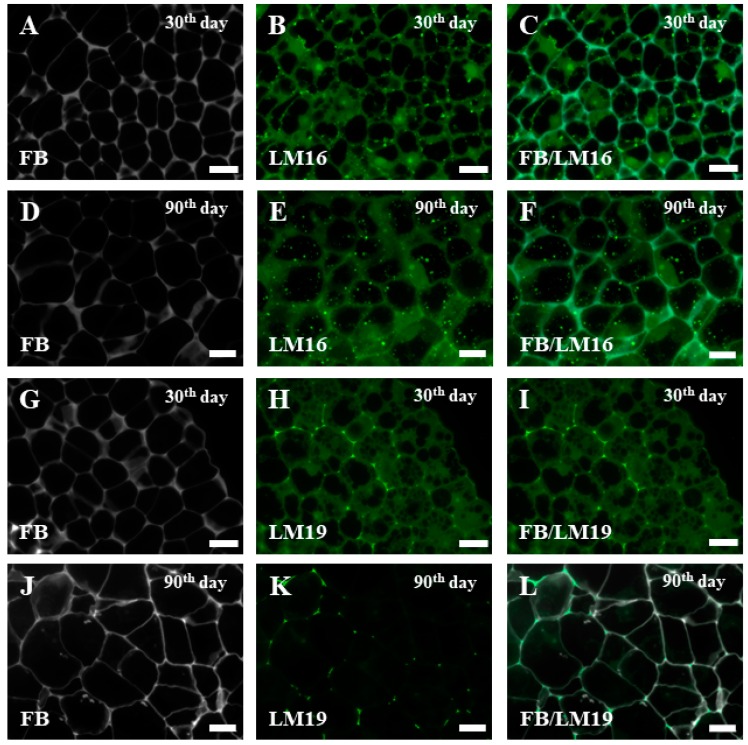
Immunolocalisation of LM16 (**A**–**F**) and LM19 (**G**–**L**) in *B. distachyon* callus. (**A**–**C**, **G**–**I**): 30th day of cultivation; (**D**–**F**, **J**–**L**) 90th day of cultivation. The greenish background on these photomicrographs is due to autofluorescence. Scale bars, 10 μm.

**Figure A5:**
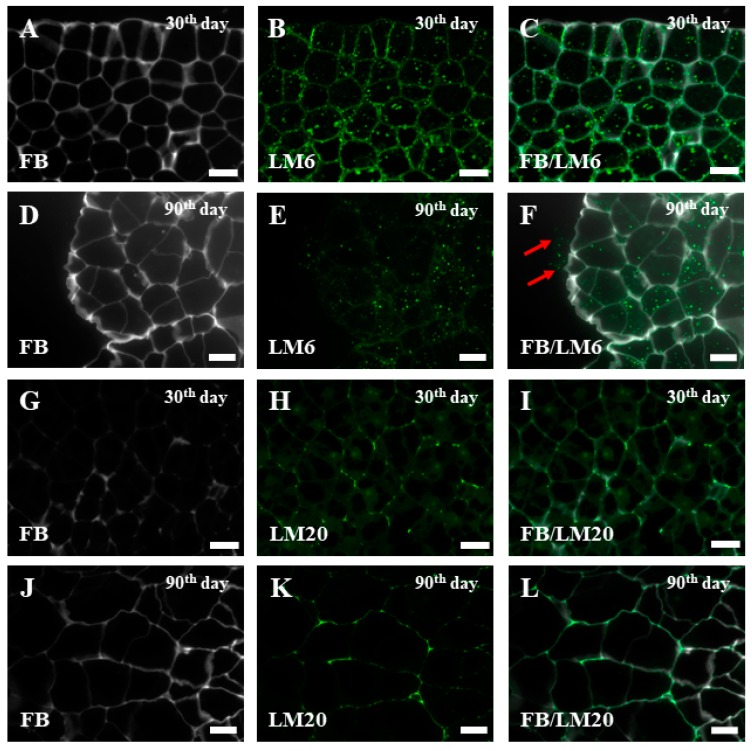
Immunolocalisation of LM6 (**A**-**F**) and LM20 (**G**–**L**) in *B. distachyon* callus. (**A**–**C**, **G**–**I**) 30th day of cultivation; (**D**–**F**, **J**–**L**) 90th day of cultivation. The red arrows indicate the presence of the epitope on the callus surface. Scale bars, 10 μm.

**Figure A6:**
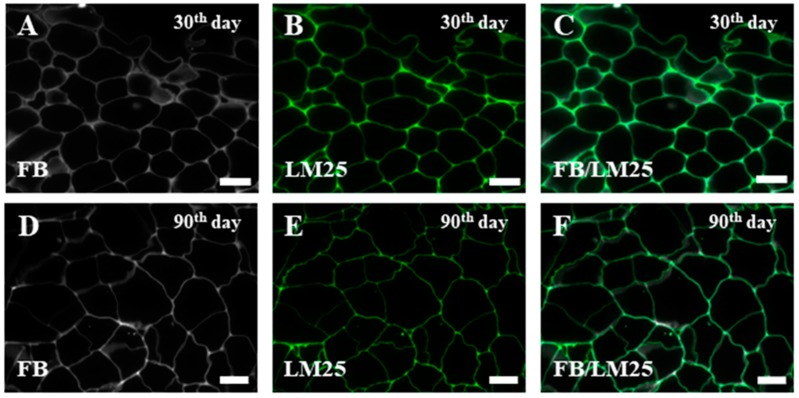
Immunolocalisation of LM25 (**A**–**F**) in *B. distachyon* callus. (**A**–**C**) 30th day of cultivation; (**D**–**F**) 90th day of cultivation. Scale bars, 10 μm.

The AGP-specific JIM13 epitope was distributed throughout the cell wall in 30-day-old calli (Figure A1A–C) but was also localised on the cell surface in 90-day-old calli, (Figure A1D–F). JIM16 epitope distribution also showed stage-related differences, being mostly present in intercellular spaces and the cell wall (Figure A1G–I) in 30-day-old calli and also accumulated in intracellular compartments in 90-day-old calli (Figure A1J–L). The LM2 epitope signal was the strongest in 30-day-old calli (Figure A2A–C), being present mostly in intracellular compartments, though some signal was detected also in the plasmalemma and rarely in the cell wall. After 90 days of culture, the signal was essentially limited to intracellular compartments (Figure A2D–F). The EXT-specific JIM11 epitope could not be detected at either time point (Figure A3A–F). The JIM12 epitope signal, weak on the 30th day (Figure A3G–I, H–red arrow), was significantly increased by the 90th day (Figure A3J–L) and localised in the cell wall at both time points. The signal from the pectin-specific LM16 epitope also significantly increased from the 30th to the 90th day of culture (Figure A4A–F) but was present in the form of dotted signal predominantly in intracellular compartments and very rarely in the cell wall. The LM19 epitope was distributed in the periclinal cell walls and in the cell corners, mostly in the second-third cell layers from the callus surface (Figure A4G-I). On the 90th day, this epitope was detected predominantly in cell corners (Figure A4J–L). In 30-day-old calli, the LM6 epitope was observed primarily in intracellular compartments, probably in the plasmalemma and in the cell wall (Figure A5A–C) but, on day 90, the signal was also detectable outside the callus cells (Figure A5D–F, F–red arrows), indicating that this epitope was secreted into the medium. LM20 was localised in the cell wall corners after 30 days of the culture (Figure A5G–I) and, after 90 days of culture, it was present in the entire cell wall (Figure A5J–K). The hemicellulose-specific LM25 antibody was detected in cell walls independently of the culture duration (Figure A6A–F).

Summarising these results over the entire culture period, immunohistochemical studies showed that: 1. AGPs decreased with the time of culture; 2. Among the extensin epitopes, the amount of JIM12 increased, but the amount of JIM11 decreased; 3. Among the pectin epitopes, arabinan-RGI did not change over time; LM19 and LM6 decreased, but LM20 increased with the time of culture; 4. Hemicelluloses remained constant.

**Figure 4:**
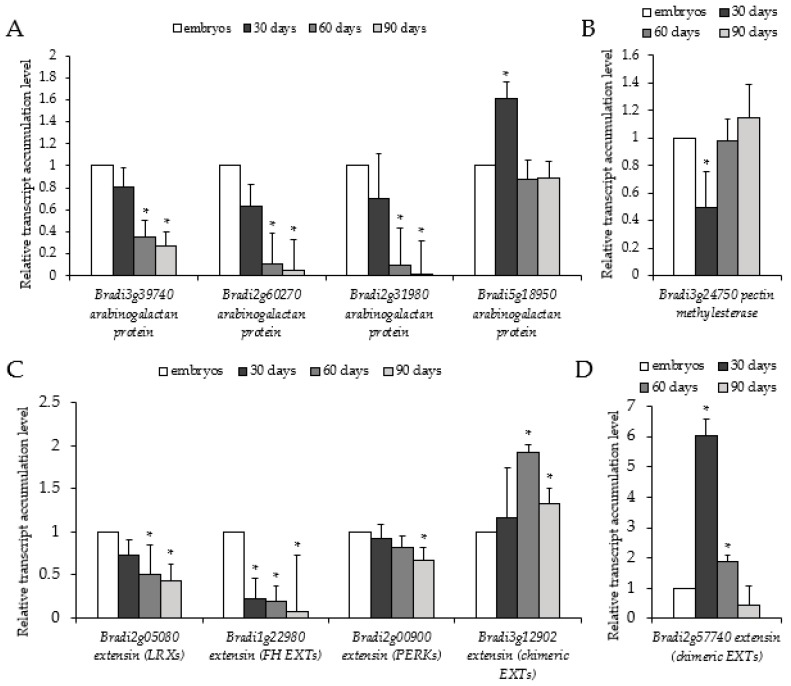
Relative transcript accumulation levels of genes associated with cell wall architecture, i.e., *AGPs* (*Bradi3g39740*, *Bradi2g60270*, *Bradi2g31980*, and *Bradi5g18950*), *EXTs* (*Bradi2g05080*, *Bradi1g22980*, *Bradi2g00900*, *Bradi3g12902*, and *Bradi2g57740*), and *PECTIN METHYLESTERASE* (*Bradi3g24750*)), on the 30th, 60th, and 90th day of cultivation. Relative transcript accumulation levels of (**A**) *AGPs*, (**B**) *EXTs*, (**C**) *PECTIN METHYLESTERASE*, (**D**) chimeric *EXT Bradi3g12902*. The relative transcript accumulation levels were normalised to an internal control (*AK437296*, gene encoding for ubiquitin) and calibrated to the control (explants, zygotic embryos); *: value is significantly different from the control (Student’s *t*-test, *p* < 0.05; *n* = 3 ± SD).

Next, we used RT-PCR to see how transcript levels related to epitope signals. Transcript levels for selected *AGPs* (*Bradi3g39740*, *Bradi2g60270*, and *Bradi2g31980*) and *EXTs* (*Bradi2g05080*, *Bradi1g22980*, and *Bradi2g00900*) decreased throughout the time period (Figure 4A,C). Unlike *AGPs* and *EXTs*, the level of pectin methylesterase (*Bradi3g24750*) increased (Figure 4B). The transcript level of *Bradi5g18950* and *Bradi2g57740*, which correspond to *AGP* and *EXT* (chimeric *EXTs*), demonstrated the highest accumulation in 30-day-old calli. *Bradi2g57740* transcript (Figure 4D) was almost 5-fold higher in 30-day-old calli compared to all analysed *EXTs* (Figure 4C). *Bradi3g12902* (chimeric *EXT*) revealed the highest accumulation on day 60. To summarise, the decreased immunolocalisation signals were broadly in accordance with the determined transcript accumulation levels.

### 2.3. Transcript Accumulation Analysis of the Genes Related to Meristem Development and Cell Division

**Figure 5:**
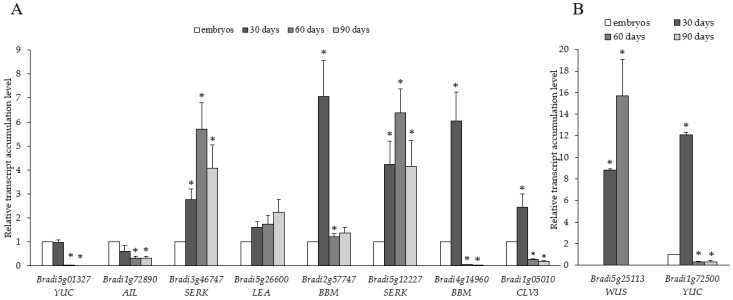
Relative transcript accumulation levels of (**A**) *YUC* (*Bradi5g01327*), *AIL* (*Bradi1g72890*), *SERK* (*Bradi3g46747* and *Bradi5g12227*), *LEA* (*Bradi5g26600*), *BBM* (*Bradi2g577747* and *Bradi4g14960*), *CLV3* (*Bradi1g05010*), and (**B**) *WUS* (*Bradi5g25113*) and *YUC* (*Bradi1g72500*) genes on the 30th, 60th, and 90th day of cultivation. The relative transcript accumulation levels were normalised to an internal control (*AK437296*, a gene encoding ubiquitin) and calibrated to the control (explants, zygotic embryos); *: value is significantly different from the control (Student’s *t*-test, *p* < 0.05; *n* = 3 ± SD).

Somatic embryogenesis involves de novo formation of new meristems. Therefore, we examined the transcript accumulation levels of genes associated with meristem development and cell division. Comparative analysis of 10 genes linked to SE suggested differences in their transcript accumulation levels across the time course examined. A detailed description of the genes studied and oligonucleotide primers used for RT-PCR is shown in Table A1. Genes such as *YUC* (*Bradi1g72500* and *Bradi5g01327*), *AIL* (*Bradi1g72890*), *BBM* (*Bradi2g57747* and *Bradi4g14960*) and *CLV3* (*Bradi1g05010*) showed similar transcript accumulation patterns that decreased from the 30th day (Figure 5). The accumulation of *SERK* transcripts (*Bradi3g46747* and *Bradi5g12227*) increased from the 30th to the 60th day and then decreased on the 90th day. Accumulation of *LATE EMBRYOGENESIS ABUNDANT* (*LEA*) transcripts (*Bradi5g26600*) increased throughout the time course. Interestingly, *WUS* (*Bradi5g25113*) transcript was detected only on the 30th and 60th day time points and was undetectable on the 90th day. The relative transcript accumulation of *WUS* (*Bradi5g25113*.1) and *YUC* (*Bradi1g72500*) genes was more than eight-fold and ten-fold higher at 30 days than in the embryos (Figure 5B).

**Figure 6:**
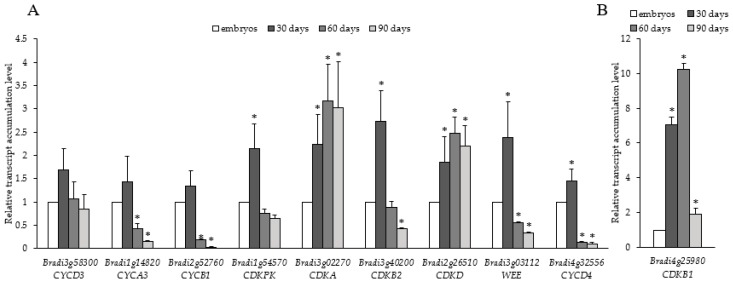
Relative transcript accumulation levels of (**A**) *CYCD3* (*Bradi3g58300*), *CYCA3* (*Bradi1g14820*), *CYCB1* (*Bradi2g52760*), *CDKPK* (*Bradi1g54570*), *CDKA* (*Bradi3g02270*), *CDKB2* (*Bradi3g40200*), *CDKD* (*Bradi2g26510*), *WEE* (*Bradi3g03112*), and *CYCD4* (*Bradi4g32556*), and (**B**) *CDKB1* (*Bradi4g25980*) on the 30th, 60th and 90th day of culture. The relative transcript accumulation levels were normalised to an internal control (*AK437296*, gene encoding ubiquitin) and calibrated to the control (explants, zygotic embryos); *: value is significantly different from the control (Student’s *t*-test, *p* < 0.05; *n* = 3 ± SD).

Ten cell cycle-related genes have mostly similar patterns of transcript accumulation. *CYCD3* (*Bradi3g58300*), *CYCA3* (*Bradi1g14820*), *CYCB1* (*Bradi2g52760*), *CDKPK* (*Bradi1g54570*), *CDKB2* (*Bradi3g40200*), *WEE* (*Bradi3g03112*), and *CYCD4* (*Bradi4g32556*) transcript levels were the highest in 30-day-old calli (Figure 6). Then, the levels decreased on the 60th and 90th day. Unlike other cell cycle-related transcripts, *CDKA* (*Bradi3g02270*) and *CDKD* (*Bradi2g26510*) showed their highest levels on the 60th and 90th day. The highest relative change in transcript level among all the cell cycle-related genes analysed was for *CDKB1* (*Bradi4g25980*) (Figure 6B), whose expression increased from the 30th to 60th day and was ten-fold higher on the 60th day of culture compared to the embryos. However, *CDKB1* transcripts then dramatically decreased on the 90th day.

## 3. Discussion

*B. distachyon* is a model plant widely used to study various aspects of cereal crop and forage grass biology [33,34], including the cytogenetic and molecular mechanisms underpinning development under both in vivo and in vitro conditions [10,35,36,37,38]. Genetic transformation technologies, critical for the discovery and validation of gene function, have been optimised for successful use in *B. distachyon* [39,40]. However, as we have demonstrated in the present work, *B. distachyon* tissue culture is also characterised by the rapid loss of embryogenic potential. The molecular mechanisms responsible for the loss of embryogenic potential remain elusive, but we show here endoploidy levels increasing in 60- and 90-day old calli, with effective elimination of the 2C DNA content peak in the latter. The indolic compounds content revealed a decrease in 60- and 90-day-old calli compared to either freshly isolated explants or 30-day-old calli. Moreover, the immunocytochemical analysis and the transcript accumulation levels of selected HRGPs and genes connected with meristem development and cell division revealed that most had significantly decreased signals or transcript accumulation levels on 60th and 90th day of culture. Conversely, transcript abundance for other genes significantly increased in 30-day-old calli, and this was correlated with an increased embryogenic state.

Endoploidy is a commonly observed phenomena that occurs during the culture of callus tissue of many species (*Arabidopsis thaliana* [41], *Urginea indica* [42], *Rubus chamaemorus* L. [43], and *Vicia faba* [44]). Long-term culture is conducive to increased level of endoploidy [41,45,46,47], which has been implicated in the reduction of the morphogenetic potential or in the regeneration of polyploid plants [45,48]. We found that increasing endoploidy from the 60th to the 90th day, including the loss of detectable 2C DNA content peaks on the 90th day of culture, correlated with loss of regenerative potential. The increase in nuclear DNA content are associated with significantly reduced regenerative capacity of callus tissue in *Musa* [49] or *Cucumis sativus* [45]. The gradual loss of embryogenic potential in *B. distachyon* callus culture could be also caused by a progressive accumulation of genomic changes, leading to the formation of non-totipotent polyploid cells. In most cases, loss of regenerative ability occurs in long-term culture (i.e., *Sorghum bicolor* [50] and *U. indica* [42]) but in other species can be observed after only weeks (i.e., hairy root culture of *Onobrychis viciaefolia* [51] and callus culture of *Pennisetum americanum* [52]). The retention of embryogenic potential can be preserved by changing media components, as it was shown in the culture of *S. bicolor* [50], *Saccharum officinarum* L. [16] and *P. americanum* [52]. The reduction or replacement of 2,4-Dichlorophenoxyacetic (2,4-D) can also be helpful in maintaining the regeneration ability. 2,4-D promotes the occurrence of chromosomal aberrations and somaclonal variation but at lower levels it can promote retention of a large pool of diploid cells with high embryogenic potential [53,54].

Changes in endogenous levels of indolic compounds are associated with SE. A preincubation period increases both free and conjugated indole-3-acetic acid (IAA), as well as indole-3-butyric acid in *Coffea canephora* [55]. The embryogenic suspensor mass of *Abies alba* contains significantly higher amounts of endogenous IAA with a medium supplemented with 2,4-D [56]. The decrease in endogenous IAA seems to be an important stimulus for the subsequent development of embryos. Hu et al. [57] demonstrated that the elevated level of endogenous auxin inhibits in vitro shoot organogenesis in citrus. In our work, we demonstrated the highest level of indolic compounds was in zygotic embryos and on the 30th day of culture. The level of these compounds significantly decreased on the 60th and 90th day of the culture. Accordingly, the level of indolic compounds is correlated with embryogenic potential. Mamedes-Rodrigues et al. [58] showed that embryogenic calli of *B. distachyon* are characterised by the highest potential for plantlet production on the 60th and 90th day of culture and detailed the metabolite profiles that highlighted an association of amino acids such as aspartic acid, asparagine, tryptophan, and glycine with embryogenic competence. Higher levels of plantlet regeneration, therefore, seem to be connected with a decreasing amount of indolic compounds, important for embryo maturation.

Association of HRGPs with morphogenesis in vitro has been previously described [59,60]. The HRGP family contains three members: highly glycosylated AGPs, moderately glycosylated EXTs, and slightly glycosylated proline-rich proteins (PRPs). Different types of AGPs seem to have different influences on SE. Some seem to stimulate SE, while others might be involved in its inhibition [28,61]. The expression of specific HRGP epitopes is correlated with SE, and some epitopes are effective markers of embryogenic cells [10,11,59]. Our immunolocalisation of specific HRGP epitopes demonstrate differences with respect to the times that coincide with *AGP* transcript accumulation. Some of the chimeric *EXTs* increased only on the 30th day of callus culture and seem to be good markers for embryogenic cells. Differential gene expression analysis of *Picea balfouriana* calli identified 431 significantly upregulated genes and 987 significantly downregulated genes in embryogenic tissues relative to non-embryogenic tissues [62]. Moreover, the authors reported the upregulation of the *AGP* genes in the embryogenic tissues. AGP epitopes in immature seeds are developmentally regulated, and the biological activity of AGPs in the formation of somatic embryos changes depending on the age of the seeds [62,63,64,65]. In an earlier study, we demonstrated the presence of AGP and EXT epitopes in the zygotic embryos of *B. distachyon* [66].

AGP levels tend to reduce as a function of culture time. AGPs are present in various plant organs including roots, stems, leaves, flowers, and seeds and have been implicated in many developmental processes, molecular interactions, plant signalling, and in embryogenesis (reviewed by [59]). Because AGPs are anchored to the cytoplasmic membrane by a glycosylphosphatidylinositol (GPI) anchor, they are good candidates for signalling molecules involved in signal exchange during in vitro culture [12,67]. The disappearance of some AGP epitopes during culture might be a manifestation of aging and of a loss of embryogenic potential. In carrot in vitro cultures, AGPs are present in embryogenic lines but undetectable in non-embryogenic or weakly embryogenic lines, supporting a correlation with SE [68].

In the primary cell wall, pectins form the matrix in which cellulose microfibrils and hemicelluloses are embedded [69]. Key pectins include homogalacturonan (HGA) and rhamnogalacturonan I (RGI). Pectin domains crosslink to each other via calcium and boron bonds [70,71]. These connections can be modified by pectin methylesterases (PMEs), which catalyse the demethylesterification of homogalacturonans [70]. Demethylesterification increases HGA capacity to crosslink via calcium ions, which causes cell wall stiffening, compaction, and enhanced cell–cell adhesion [72,73]. PMEs from bean hypocotyls may participate in cell wall degradation [74]. PMEs can change the pH and consequently activate other cell wall-degrading enzymes, thus facilitating cell expansion, growth, and separation [75,76]. In the present work, we found increased presence of LM20 epitopes as well as increased transcript accumulation levels of *PECTIN METHYLESTERASE*. This was associated with a decrease in embryogenic potential. Experimental manipulation of PMEs during embryogenesis is required to evaluate potential functional roles.

The correct progression of SE depends on several transcription factors as well as other proteins [77]. For example, *LEC* and *BBM* genes are embryo marker genes that play important roles in cell proliferation and embryogenesis in *A. thaliana* [78,79]. These genes are expressed in proliferating epidermal and subepidermal cells from which somatic embryos develop, and these clusters of cells are required for embryo identity. *BBM* is a member of the *AIL* clade of AP2/ERF transcription factors, and these transcription factors can convert somatic cells into totipotent embryogenic cells [80]. The mechanism of such conversion could be similar in *B. distachyon*, as we have found the highest transcript accumulation level of *BBM* and *AIL* genes on the 30th day of culture when histological analysis demonstrated the presence of the highest number of embryogenic masses. Transient expression of *BBM* could help to promote somatic embryogenesis in *B. distachyon*.

*SERK*, *WUS*, and *CLV3* are meristem organisation genes that play a key role in the induction of embryogenesis, and their expression increases during the early stages [81,82]. Transcriptional activity of the *WUS* gene in *A. thaliana* cells is sufficient to maintain meristematic cells in the shoot and flower meristems [83]. In our research, the *WUS* gene was expressed only on the 60th and 90th day of culture which coincides with the highest potential of plantlet production [58]. *WUS* activates the expression of *CLV3*, while *CLV3* inhibits the expression of the *WUS* gene, and this feedback system helps to maintain a constant pool of stem cells. The *WUS* gene also plays a key role in embryogenesis, facilitating the transfer of cells from somatic identity to an embryogenic fate, and is involved in the maintenance of embryogenic stem cells [84,85].

The *SERK* and *LEA* genes are associated with SE in various species, such as *Dactylis glomerata* and *A. thaliana* [86,87,88,89,90]. In our experiments, we found the highest transcript accumulation level of *SERK* genes on the 60th day. On the 90th day of culture, the transcript accumulation level was similar to the one on the 30th day of culture. It should be noted that the function of *SERK* genes is not restricted to SE. Along with the *LEA* genes, they are expressed under the influence of biotic and abiotic stress [82,91,92]. Callus aging coincides with the production of reactive oxygen species (ROS), stress molecules that are correlated with higher expression levels of *SERK* genes. In barley, the expression of *HvSERK2* was rapidly induced by hydrogen peroxide treatment [93].

IAA is involved in the proper expression of SE-related genes. For example, in *C. canephora,* an increase in IAA and indole-3-butyric acid (IBA) is associated with higher expression of the *YUC* and *TRYPTOPHAN AMINOTRANSFERASE* (*TAA*) genes [55]. Wójcikowska et al. [94] demonstrated that *YUCCA* and *TAA1* are involved in the tryptophan-dependent IPA–YUC auxin biosynthesis pathway and are associated with SE induction. A similar situation occurs in *B. distachyon*, where the increase in *YUC* (*Bradi1g72500* and *Bradi5g01327*) transcript accumulation level is accompanied by high levels of indolic compounds on the 30th day of culture.

CDKs and cyclins are major regulators of the cell cycle, and their correct expression and activity are crucial for both cell differentiation and cell proliferation. We found higher transcript accumulation levels of all analysed *CYC* and *CDK* genes on the 30th day, which seems to be connected with normal cell cycle progression. However, the transcript accumulation level of the most analysed *CYCs* and *CDKs* decreased on the 60th and 90th day. Such differences seem to be connected with diversion of older cultures into the endoreduplication program, which requires lower CDK kinase activity [95]. These changes were demonstrated using flow cytometry, since we observed increased DNA content in the analysed probes. A key step for the inhibition of CYC/CDK activity is the upregulation of *CYCLIN KINASE INHIBITORS* (*CKIs*) during the endoreduplication process [95,96]. The overexpression of the R2 cDNA, which encodes the rice CDK-activating kinase, in tobacco leaves triggered callus formation without requiring exogenous cytokinins [97]. Moreover, R2 expression in the later stages did not prevent cell differentiation and progression to plant organs. Thus, it seems that CDKs control the differentiation fate of the cells during organogenesis. In *Cocos nucifera, CDKA* transcripts increase during embryogenic callus formation [8]. The expression level of *CDKA* decreases in germinated somatic embryos. In our research, we observed the increase of the *CDKA* transcript accumulation level from the 30th through the 60th and 90th day of embryogenic callus culture.

## 4. Materials and Methods

### 4.1. Plant Material Growth and In Vitro Culture Conditions

Immature zygotic embryos of *B. distachyon* reference line Bd21 were used as explants for embryogenic callus induction. Callus induction medium (CIM, pH 5.8) contained MS salts, vitamins, 30 g/L sucrose, 2.5 mg/L 2,4-D, and 8 g/L Select Agar. Petri dishes with embryos were incubated at 28 °C in the dark for embryogenic callus induction and subsequent callus culture. Calli with embryogenic complexes were transferred to fresh CIM every three weeks. Callus analyses were performed on the 30th, 60th, and 90th day after explant culture. All images were taken using a dissecting microscope SMZ 1500 (Nikon, Shinagawa, Tokyo, Japan) equipped with a digital camera DS-U2 (Nikon, Shinagawa, Tokyo, Japan).

### 4.2. Flow Cytometry

Flow cytometry was used to analyse changes in the ploidy level in *B. distachyon* callus. To determine the DNA content, zygotic embryos, embryogenic callus, and non-embryogenic parts were used on the 30th, 60th, and 90th day of culture. The samples were analysed using a CyFlow Space flow cytometer (Sysmex, Kobe, Japan) as in Wolny et al. [38].

### 4.3. Indolic Compounds Estimation

In order to estimate the IAA content, a colorimetric technique according to Bric et al. [98] was applied to detect indolic compounds. Explants—freshly isolated zygotic embryos—were placed in media designed to promote callus and SE formation and were analysed after 30, 60, and 90 days of culturing. In total, 100 mg of plant material was transferred to mortars containing 2 mL of 10 × PBS and homogenised. The solution was centrifuged (25 min; 18,000× *g*), and 2 mL of supernatant was mixed with 100 µL of 10 mM orthophosphoric acid and 4 mL of Salkowski’s reagent (150 mL H_2_SO_4_; 250 mL ddH_2_O; 7.5 mL 0.5 M FeCl_3_ × 6H_2_O). The pink colour developed after a 30 min incubation at room temperature, and the absorbance was read at 530 nm. The concentration of indolic compounds was determined by using a calibration curve of pure IAA as the standard, following linear regression analysis. Each analysis was carried out in three replicates.

### 4.4. Histological and Immunochemical Analysis

To determine the chemistry of the cell wall, a set of monoclonal antibodies that had been raised against specific cell wall epitopes such as AGPs (antibodies JIM13, JIM16, LM2), EXTs (JIM11 and JIM12), pectins (LM6, LM16, LM19, LM20), and hemicelluloses (LM25) was used. The references and information on the antibodies are shown in Table 1. The detailed procedure for histological sections observation and immunochemical analysis was as in Betekhtin et al. [10].

### 4.5. RT-PCR

In order to characterise the transcript accumulation level of selected genes, RT-PCR using a LightCycler^®^ 480 SYBR Green I Master in a LightCycler^®^ 480 Real-Time PCR System was used. The total RNA was isolated from immature zygotic embryos and from the callus cultivated for 30, 60, and 90 days. The primers used in this research are shown in Table A1. The genes encoding extensins with their division in classes was based on Liu et al. [99]. *AGP* genes were selected by keyword search in the Phytozome database (https://phytozome.jgi.doe.gov/pz/portal.html). The detailed procedure for RT-PCR was as in Betekhtin et al. [100].

## 5. Conclusions

Here, we describe the gradual decrease of the embryogenic potential in calli of *B. distachyon*. We show that numerous cell wall components undergo remodelling and that most analysed epitopes decrease in abundance as the calli lose competence. Loss of embryogenic potential is also accompanied by significant changes in the expression of genes connected with the cell wall, meristem development, and cell cycle. Moreover, we found an increase in the endoreduplication processes after 60 and 90 days of culture, which consequently leads to the disappearance of cells with 2C DNA content on 90th day. To understand more precisely the function of HRGPs and pectins during the formation of the embryogenic mass, specific inhibitors could be considered in future studies. CRISPR/Cas9-based targeted mutagenesis of selected cell wall, meristem development, and cell cycle genes may help to better understand the mechanisms governing the gradual loss of embryogenic potential in the tissue culture using *B. distachyon* as a model for other recalcitrant monocotyledonous species.

## Figures and Tables

**Table 1:** Antibodies used for immunocytochemistry, cognate epitopes, and references.

Antibody	Epitope	References
*Arabinogalactan proteins (AGPs)*		
JIM13	βGlcA1->3αGalA1->2Rha	[25,26,27]
JIM16	AGP glycan	[25,26,27]
LM2	β-linked GlcA	[26,28]
*Extensins*		
JIM11	Extensin	[25,29]
JIM12	Extensin	[29]
*Pectins*		
LM6	αAra1-5αAra1-5αAra1-5αAra1-5Ara	[30]
LM16	Processed arabinan—rhamnogalacturonan (RG)-I domain	[31,32]
LM19	α-GalA(1-4)α-GalA(1-4)α-GalA(1-4)α-GalA	[31,32]
LM20	Homogalactouronan	[32]
*Hemicelluloses*		
LM25	Xyloglucan	[30]

## Appendix A

**Table A1:** Oligonucleotide primers used for RT-PCR reaction with the relevant description of the genes.

Genes	Description of the Genes	Primer Sequence (5′–3′)
*AK437296*	*ubiquitin*	pF–TCAAAATGCAAGAACGCAAA
		pR–TCCACACTCCACTTGGTGCT
***Cell cycle***		
*Bradi1g54570*	*protein kinase binding, CDKPK*	pF–TTGTGAAGAGGTTCGCGGATGC pR–CCTTCAAGCTCCTTCAGATCC
*Bradi3g02270*	*cyclin-dependent kinase (CDK), subfamily CDKA*	pF–CGAGAAGGTGGAGAAGATCG
		pR–CGATGGTCTCGTTGGTGTAG
*Bradi4g25980*	*cyclin-dependent kinase (CDK), subfamily CDKB1*	pF –AAGTGTACAAGGCGCAGGAC
		pR–ATCCCTTCGTCGTCCATCTC
*Bradi3g40200*	*cyclin-dependent kinase (CDK), subfamily CDKB2*	pF–AGGGCCAGACCATCCTCTAC
		pR–GGATCTTCTCGTGGTTCTGG
*Bradi2g26510*	*cyclin-dependent kinase (CDK), subfamily CDKD*	pF–ACAATGGCCAGACATGGTTT
		pR–CCATTGGAAACAATGAACGA
*Bradi1g14820*	*CYCLIN, subfamily CYCA3*	pF–ATCCTTGTTGACTGGCTCGT
		pR–CGGTCGATGTAGGAGATGGT
*Bradi2g52760*	*CYCLIN, subfamily CYCB1*	pF–GTCCTGGGAAAGCAGAAGGT
		pR–GGACGTTGACGACGTTGC
*Bradi3g58300*	*CYCLIN, subfamily CYCD3*	pF–AGCTGTGACTGCTTGCTCAT
		pR–GATAAGGTCAGACGAGCGGG
*Bradi4g32556*	*CYCLIN-D4-1-RELATED, CYCD4*	pF–CTTGTCTGTAGCGGCCAAGA
		pR–CTGGATCGTCATGGCTTCGA
*Bradi3g03112*	*wee1-like protein kinase (WEE)*	pF–AGGATTTCTTCTGCACCCCG
		pR–GGAGATTTGGGGCAAGGGAT
***Meristem development***		
*Bradi1g72500*	*flavin-dependent monooxygenase, * *YUCCA (YUC)*	pF–ACCGTCCAGTGGTACAAGTTTGAG pR–AGCTTGCAGTAGTCCAAGGAGTG
*Bradi5g01327*	*lavin-dependent monooxygenase, * *YUCCA (YUC)*	pF–CACCGGCTACAAGAGCAATGTTCC pR–ATATCCGTCCTTGCCGAACAAGCC
*Bradi1g72890*	*AINTEGUMENTA-LIKE (AIL)*	pF–TGTACCTTGGCACCTTCAGCAC pR–ATGCGCTTCACGTCGTACTTGC
*Bradi5g26600*	*LATE EMBRYOGENESIS ABUNDANT (LEA)*	pF–GTGAATTGTCCGGCGTTGCTTAC pR–ACCACCAGCAACATCACCATAGC
*Bradi3g46747*	*SOMATIC EMBRYOGENESIS RECEPTOR LIKE KINASE (SERK)*	pF–CTGCTGAACTGGGCAACCTAAC pR–CATGCTGTTGTTGTTCAGACGAAG
*Bradi5g12227*	*SOMATIC EMBRYOGENESIS RECEPTOR LIKE KINASE (SERK)*	pF–AACTCCTGTGGCACAAGGTGACTC pR–GCAAATCCAATTGCTGGGACTGC
*Bradi2g57747*	*BABY BOOM (BBM)*	pF–GATCTCTACTTGGGCACTTTCAGC pR–AGTTAGTGACGGCGTTCAGC
*Bradi5g14960*	*BABY BOOM (BBM)*	pF–GAGGCTCATCTCTGGGACAATAGC
		pR–CTCCTTGTCATAGCCACCTTGC
*Bradi5g25113*	*WUSCHEL (WUS)*	pF–GGATCGAGGGGAAGAACGT
		Pr–TTGTTGTTGGGGGAGACGTC
*Bradi1g05010*	*CLAVATA 3 (CLV3)*	pF–AAGATCGCCTGGTCGAGCATAG pR–TTCTGCCTCATCAGTCGGAAGC
***Cell wall development***		
*Bradi3g39740*	*arabinogalactan protein*	pF–GTACTATTCCCTGGCGGAGTTC pR–CCATGTTGTCGGTGAGGTTGAG
*Bradi2g60270*	*arabinogalactan protein*	pF–AGCAGAGCAATCCTCTAGTAGC pR–TGGGTTCTTCTCGCCATTGTTA
*Bradi5g18950*	*arabinogalactan protein*	pF–AATAAAGGGAAGTCACCGTCGC pR–CCGTTCTTCTTGTCATGGACCT
*Bradi2g31980*	*arabinogalactan protein*	pF–AGTACCCCCTTCGGTTTCGT pR–TGGTCGATGGACGATGCGTC
*Bradi2g57740*	*extensin (chimeric EXT)*	pF–AATACAGCGTGGGCATCACA pR–AGTCAGATCCTCCTGGTGCT
*Bradi3g12902*	*extensin (chimeric EXT)*	pF–CATCTGGACCTGCCAATGGT pR–TCCCAGTTTTGGAGTCTCGC
*Bradi2g05080*	*leucine-rich repeat extensin (LRXs)*	pF–CTCCGGTTCAACGAGTTCGAG pR–CGATGTTATCCGGGAGGTTGAA
*Bradi2g00900*	*proline-rich extensin-like receptor kinases (PERKs)*	pF–TAACTTTGAGGCACAGGTTGCT pR–AGCCATGTATCCAAAAGTCCCC
*Bradi1g22980*	*formin-homolog extensin (FH EXTs)*	pF–CAGCAGAGCCTGTTGCTTGAC pR–TTCTAGGTTTCCGTGCATGAGT
*Bradi3g24750*	*pectin methylesterase*	pF–CAAGACCAAGAACTTCGTCACC pR–ATAACCCTGGATGTCTGGTCGT
